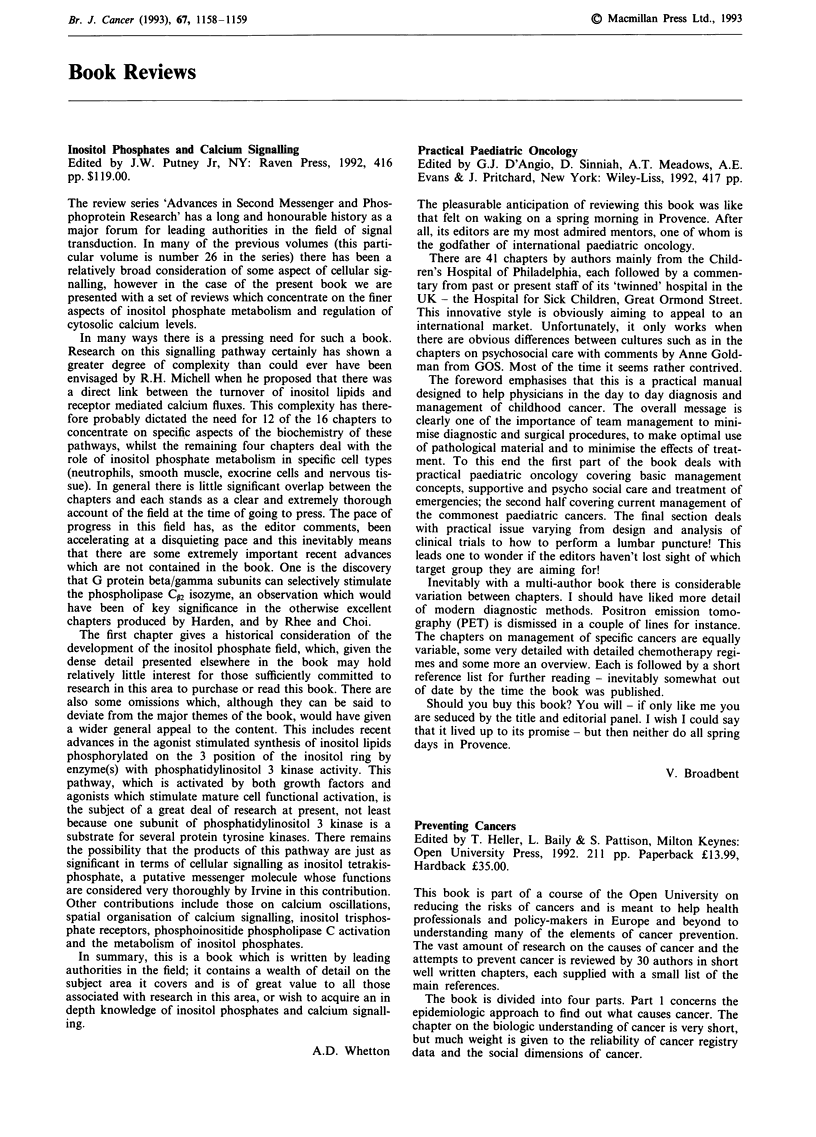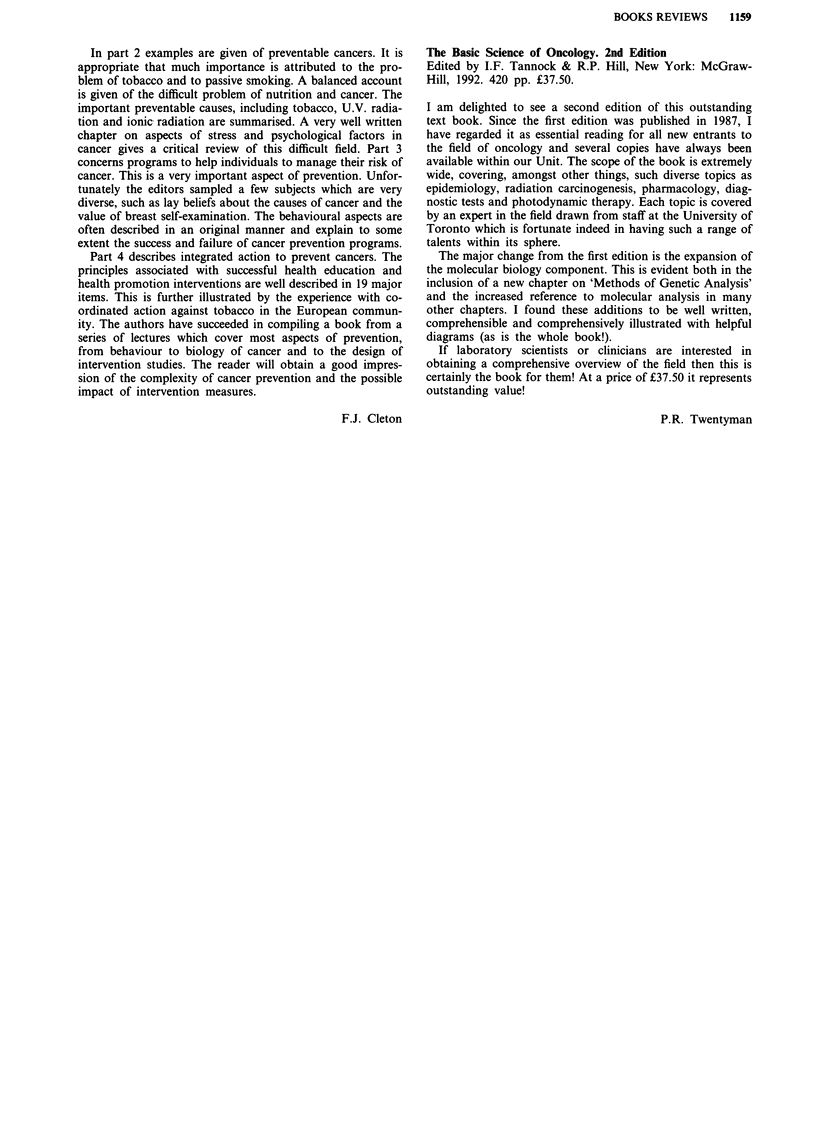# Preventing Cancers

**Published:** 1993-05

**Authors:** F.J. Cleton


**Preventing Cancers**
Edited by T. Heller, L. Baily & S. Pattison, Milton Keynes: Open University Press, 1992. 211 pp. Paperback £13.99, Hardback £35.00.

This book is part of a course of the Open University on reducing the risks of cancers and is meant to help health professionals and policy-makers in Europe and beyond to understanding many of the elements of cancer prevention. The vast amount of research on the causes of cancer and the attempts to prevent cancer is reviewed by 30 authors in short well written chapters, each supplied with a small list of the main references.

The book is divided into four parts. Part 1 concerns the epidemiologic approach to find out what causes cancer. The chapter on the biologic understanding of cancer is very short, but much weight is given to the reliability of cancer registry data and the social dimensions of cancer.

In part 2 examples are given of preventable cancers. It is appropriate that much importance is attributed to the problem of tobacco and to passive smoking. A balanced account is given of the difficult problem of nutrition and cancer. The important preventable causes, including tobacco, U.V. radiation and ionic radiation are summarised. A very well written chapter on aspects of stress and psychological factors in cancer gives a critical review of this difficult field. Part 3 concerns programs to help individuals to manage their risk of cancer. This is a very important aspect of prevention. Unfortunately the editors sampled a few subjects which are very diverse, such as lay beliefs about the causes of cancer and the value of breast self-examination. The behavioural aspects are often described in an original manner and explain to some extent the success and failure of cancer prevention programs.

Part 4 describes integrated action to prevent cancers. The principles associated with successful health education and health promotion interventions are well described in 19 major items. This is further illustrated by the experience with coordinated action against tobacco in the European community. The authors have succeeded in compiling a book from a series of lectures which cover most aspects of prevention, from behaviour to biology of cancer and to the design of intervention studies. The reader will obtain a good impression of the complexity of cancer prevention and the possible impact of intervention measures.

F.J. Cleton